# Supplementary material for: K isotopes trace temporal silicate weathering intensity
Source: Nat Commun. 2025 Dec 9;17:393. doi: 10.1038/s41467-025-67085-w (PMC12796489; doi:10.1038/s41467-025-67085-w)
Supplement: Supplementary file 1 — Supplementary Information [file 41467_2025_67085_MOESM1_ESM.pdf]

Inventory of Supplementary Information

Supplementary Methodology

Methodology S1. Endmember calculations.

Methodology S2. K fluxes in the Yellow River.

Supplementary Figures

Figure S1. Map of the Yellow River drainage basin, showing major tributaries and the Longmen hydrological station.

Figure S2. Dissolved K^+ flux versus the weekly physical erosion rate (PER).

Figure S3. Compilation of published $\delta^{41}K$ data for major reservoirs on the Earth.

Figure S4. Total K export in the middle Yellow River through time, partitioned into dissolved K (K_{rw}) and suspended particulate K (K_{SPM}).

Figure S5. $\delta^{41}K_{rw}$ versus suspended particulate matter (SPM) concentration.

Figure S6. Cross plots between $\delta^{41}K_{rw}$ values and K^+ sourced from (A) silicates, (B) carbonates, and (C) evaporites.

Figure S7. Dissolved K/Sr ratios from January to December 2013.

Figure S8. Cross plots of $\delta^{41}K_{rw}$ versus (A) dissolved K flux and (B) chemical weathering rate.

Figure S9. Cross plot between $\delta^{7}Li_{rw}$ and $\delta^{41}K_{rw}$.

Figure S10. Cross plot between $\delta^{138}Ba_{rw}$ isotopes and $\delta^{41}K_{rw}$.

Figure S11. Cross plot between Si and K^+ concentrations, normalized to Na*.

Figure S12. Cross plot between $\delta^{26}Mg_{rw}$ and $\delta^{41}K_{rw}$.

Supplementary Tables

Table S1. Dissolved K^+ concentrations and $\delta^{41}K_{rw}$ data and associated parameters.

Table S2. Major ions and $\delta^{41}K$ of rainwater, agricultural sewage, and groundwater collected from the Longmen hydrological station.

Table S3. K concentrations and $\delta^{41}K$ compositions of the sequential extracted

loess fractions from Lingtai.

Table S4. Saturation indices (SI) of selected aluminosilicates calculated using PHREEQC.

Supplementary references

Methodology S1. Endmember calculations

The concentrations of NO_3^-, Cl^-, SO_4^{2-}, Na^+, and Mg^{2+} of the river water samples were used to calculate the endmembers. It is assumed that NO_3^- is solely derived from anthropogenic input (e.g., agriculture), with Cl/Na = 4 ± 1, NO_3/Na = 7 ± 3, and SO_4/Na = 1 according to Zhang et al. (2015)[2]. The ions contributed by anthropogenic input to the river water can then be calculated by Eqs. S1 to S4 below.

$$[NO_3^{\ -}]_{anth} = [NO_3^{\ -}]_{rw} \qquad \text{(S1)}$$

$$[Na^+]_{anth} = [NO_3^{\ -}]_{anth} \times \left(\frac{[Na\ \ ]}{[NO_3\ \ ]}\right)_{anth} \qquad \text{(S2)}$$

$$[SO_4^{\ 2-}]_{anth} = [Na^+]_{anth} \times \left(\frac{[SO_4\ \ ]}{[Na\ \ ]}\right)_{anth} \qquad \text{(S3)}$$

$$[Cl^-]_{anth} = [Na^+]_{anth} \times \left(\frac{[Cl\ \ ]}{[Na\ \ ]}\right)_{anth} \qquad \text{(S4)}$$

The remaining Cl^- and SO_4^{2-} after anthropogenic correction are derived from rainwater and evaporites, which can be separated when the compositions of rainwater and evaporites are fixed. The rainwater endmember is reported in Zhang et al. (2015)[2] (Cl/Na = 1.17, SO_4/Na = 0.06) and the evaporite endmember is represented by the results of the loess leaching experiment (Cl/Na = 0.32 ± 0.02, SO_4/Na = 0.35 ± 0.02, 2 s.d.)[1]. These two endmembers have distinct Cl/SO_4 ratios, which allows accurate calculations of endmember mixing for the river waters in this study.

$$[SO_4{}^{2-}]_{rw} - [SO_4{}^{2-}]_{anth} = [Cl^-]_{rain} \times \left(\frac{[SO_4\ ]}{[Cl\ ]}\right)_{rain} + [Cl^-]_{evap} \times \left(\frac{[SO_4\ ]}{[Cl\ ]}\right)_{evap} \quad \text{(S5)}$$

$$[Cl^-]_{rw} - [Cl^-]_{anth} = [Cl^-]_{rain} + [Cl^-]_{evap} \quad \text{(S6)}$$

The $[\mathrm{Cl}^-]_{rain}$ and $[\mathrm{Cl}^-]_{evap}$ can be solved using Eqs. S5 and S6. Thus, the contents of Na^+ and Mg^{2+} from these two endmembers can then be calculated according to the Cl/Na and Mg/Na ratios of the endmembers, using Eqs. S7 to S10.

$$[Na^+]_{rain} = [Cl^-]_{rain} \times \left(\frac{[Na\ ]}{[Cl\ ]}\right)_{rain} \quad \text{(S7)}$$

$$[Na^+]_{evap} = [Cl^-]_{evap} \times \left(\frac{[Na\ ]}{[Cl\ ]}\right)_{evap} \quad \text{(S8)}$$

$$[Mg^{2+}]_{rain} = [Na^+]_{rain} \times \left(\frac{[Mg\ ]}{[Na\ ]}\right)_{rain} \quad \text{(S9)}$$

$$[Mg^{2+}]_{evap} = [Na^+]_{evap} \times \left(\frac{[Mg\ ]}{[Na\ ]}\right)_{evap} \quad \text{(S10)}$$

Carbonates dominate the weathering source of Mg^{2+} in the river water[3], so that the carbonate contribution can be estimated from Eq. S11. Then, the Na^+ supplied by carbonate weathering can be quantified using the Mg/Na ratio of the carbonate fraction of the loess leaching experiment in Eq. S12.

$$[Mg^{2+}]_{carb} = [Mg^{2+}]_{river} - [Mg^{2+}]_{rain} - [Mg^{2+}]_{evap} \quad \text{(S11)}$$

$$[Na^+]_{carb} = [Mg^{2+}]_{carb} \times \left(\frac{[Na\ ]}{[Mg\ ]}\right)_{carb} \quad \text{(S12)}$$

These calculations may overestimate the Mg^{2+} and Na^+ contributed by

carbonate weathering. However, since the results of the carbonate contribution to the Na^+ and K^+ budgets are low, this approximation will not influence the conclusions. After the corrections for the anthropogenic, rain, evaporite, and carbonate inputs, the remaining Na^+ is attributed to silicate weathering according to Eq. S13, as suggested by Zhang et al. (2015)[2].

$$[Na^+]_{sil} = [Na^+]_{rw} - [Na^+]_{anth} - [Na^+]_{rain} - [Na^+]_{evap} - [Na^+]_{carb} \quad \text{(S13)}$$

Since Na^+ is a conservative ion in river water, the initial K^+ concentration of the stream water before any removal of K^+ into secondary precipitates can be estimated based on the Na^+ contribution and the estimated K/Na ratio of each endmember (Fig. 3).

Methodology S2. K fluxes in the Yellow River

Rivers export terrestrial K to the oceans either in the dissolved load (K_{rw}), or as solids (K_{SPM}). The equations S14 and S15 below were employed to determine the proportions of these two major forms of riverine K[4-6]:

$$K_{rw}\ (\%) = \frac{K_{rw}\ flux}{K_{rw}\ flux + K_{SPM}\ flux} \times 100 \qquad (S14)$$

$$K_{SPM}\ (\%) = 100\% - K_{rw} \qquad (S15)$$

where the K_{rw} and K_{SPM} are given in percentage of the total K export (Fig. S4). The K_{rw} flux is the product of [K^+] and the corresponding weekly average water discharge (*Q_w),* as gauged (generally three times per day) at the sampling site. Similarly, the K_{SPM} flux is the product of the [K] in the SPM and the corresponding weekly average SPM flux.

In the middle Yellow River in 2013, K was primarily transported by the solid load (averaging ~60%), with a higher proportion of K being transported as solids during the monsoon season, at typically ~95.0% and up to 99.7 % (Fig. S4). During the ice-melting event interval in March, the fraction of K transported as solids also increased dramatically up to ~95% (Fig. S4). Approximately 45% of the total annual dissolved K^+ was transported during the monsoon season (3.5 months), and the 4-day storm event accounted for 3% of the total annual dissolved K^+ flux (Fig. 1). Note that the above estimates of K partitioning between the dissolved and SPM fractions do not account for potential variability in the solid K concentrations and SPM content with water column depth, although the middle Yellow River water appears homogeneous with respect to

Li, Ba, and Mg concentrations and isotopes in the SPM with water column depth[6, 7]. If such homogeneity is also applicable to K, our estimated K_{SPM} flux is likely to be accurate, albeit with some uncertainty due to unquantified variability of the SPM concentrations with column depth.

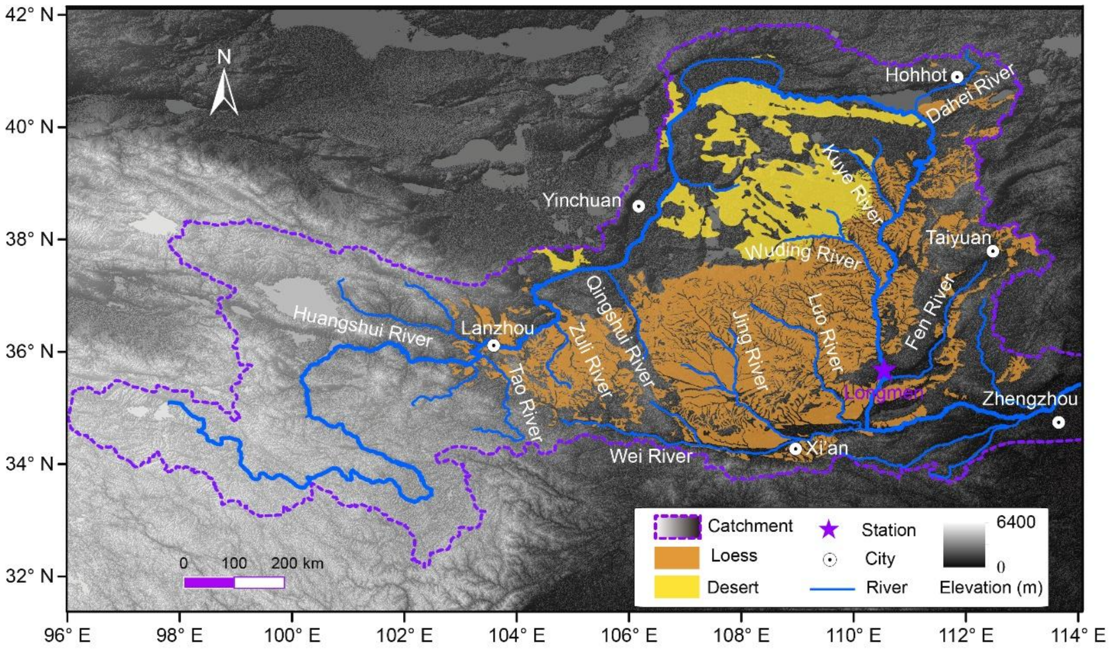

Figure S1 Map of the Yellow River drainage basin, showing major tributaries and the location of the Longmen hydrological station. Loess and desert dominate within the upper and middle reaches of the Yellow River basin. Modified from Gou et al. (2023)[1].

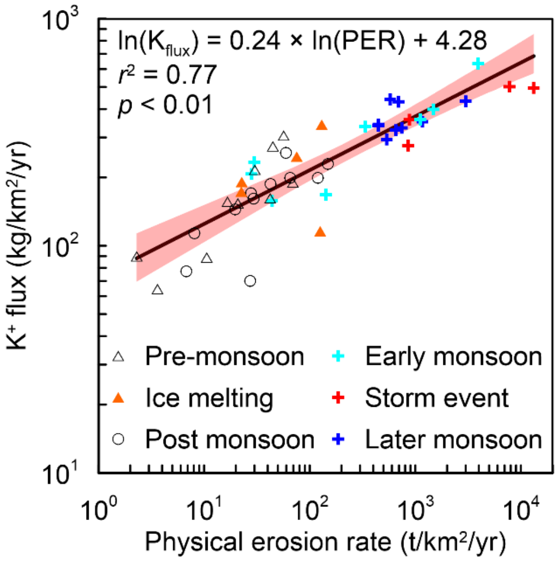

Figure S2 Dissolved K^+ flux versus the weekly physical erosion rate (PER; Zhang et al. (2015)[2]) in the middle Yellow River, showing a positive relationship.

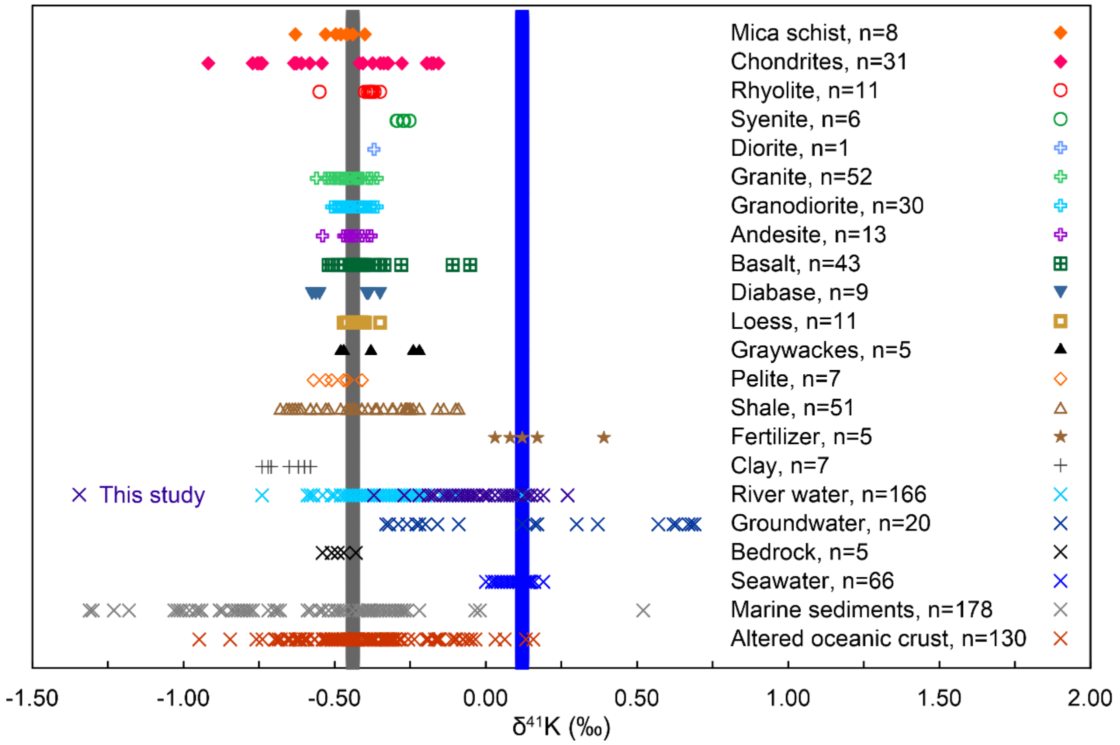

Figure S3 Compilation of published $\delta^{41}K$ data for the major reservoirs on the Earth. The blue and grey vertical bands represent the average $\delta^{41}K$ values of seawater (SW, 0.12‰, Wang et al., 2020) [8] and upper continental crust (UCC, -0.44‰, Huang et al., 2020) [9], respectively. The dark blue river water data is from this study. Note that the $\delta^{41}K$ value of MIL 13004 (chondrite) is excluded because no theory can presently explain that data (Jiang et al., 2019) [10] and it is not relevant to terrestrial weathering processes. Data sources: Altered oceanic crust (Hu et al., 2020; Santiago Ramos et al., 2020) [11, 12]; Marine sediments (Xu et al., 2019; Hu et al., 2020) [11, 13]; Seawater (Hu et al., 2018; Xu et al., 2019; Wang et al., 2020) [8, 13, 14]; Bedrock (Li et al., 2019, 2022) [15, 16]; Groundwater (Ji et al., 2024) [17]; River water (Li et al., 2019, 2022, 2023; Wang et al., 2021) [15, 16, 18, 19]; Clay (Li et al., 2019) [16]; Fertilizer (Li et al., 2022; Morgan et al., 2018) [20, 21]; Shale (Xu et al., 2019; Huang et al., 2020) [9, 13]; Pelite (Huang et al., 2020) [9]; Graywackes (Huang et al., 2020) [9]; Loess (Huang et al., 2020) [9]; Diabase (Xu et al., 2019) [13]; Basalt (Huang et al., 2020; Morgan et al., 2018) [9, 21]; Andesite (Xu et al., 2019; Huang et al., 2020) [9, 13]; Granodiorite (Xu et al., 2019; Huang et al., 2020; Mogran et al., 2018) [9, 13, 21].; Granite (Xu et al., 2019; Huang et al., 2020) [9, 13]; Diorite (Huang et al., 2020) [9]; Syenite (Xu et al., 2019)

[13]; Rhyolite (Xu et al., 2019) [13]; Chondrites (Jiang et al., 2020; Zhao et al., 2020) [10, 22]; Mica schist (Xu et al., 2019; Huang et al., 2020; Mogran et al., 2018) [9, 13].

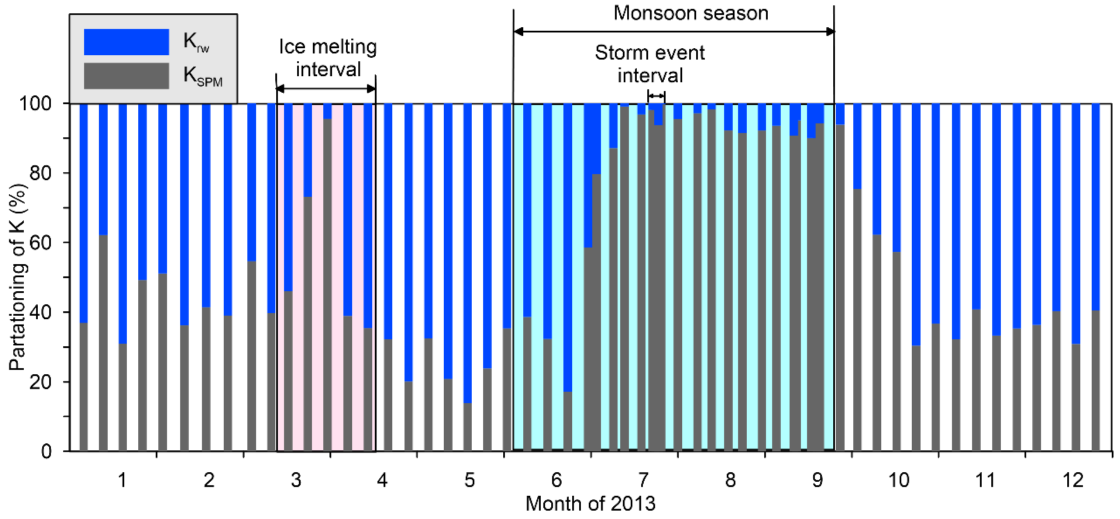

Figure S4 Total K export in the middle Yellow River through time partitioned into dissolved K^+ export (K_{rw}, blue, mean ~40%) and export as solids in suspension (K_{SPM}, grey, mean ~60%). The monsoon season had the highest proportion of K transported via solids (up to 99.7%), with K transport via solids also increasing significantly during the ice-melting and storm event intervals. See supplementary text (Methodology S2) for the calculations.

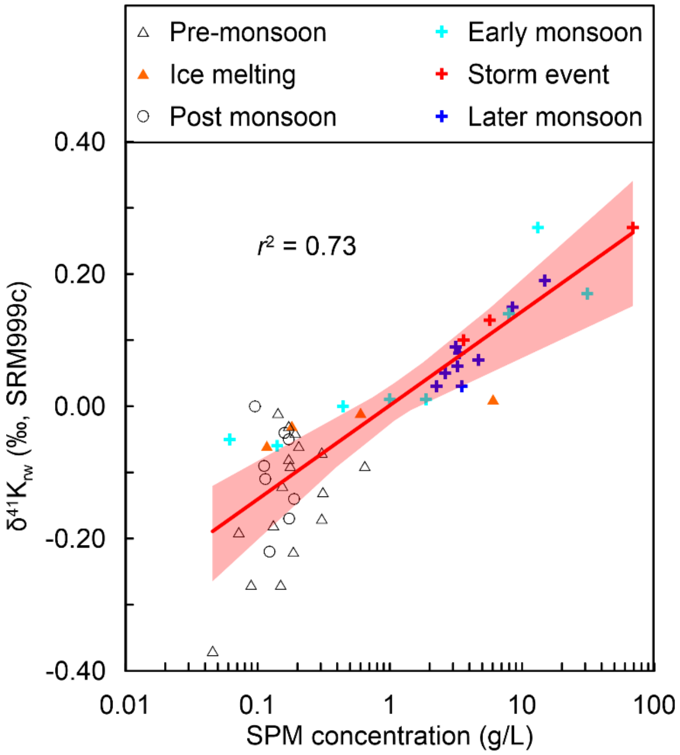

Figure S5 $\delta^{41}K_{rw}$ values versus suspended particulate matter (SPM) concentrations (on a logarithmic scale), showing a positive relationship.

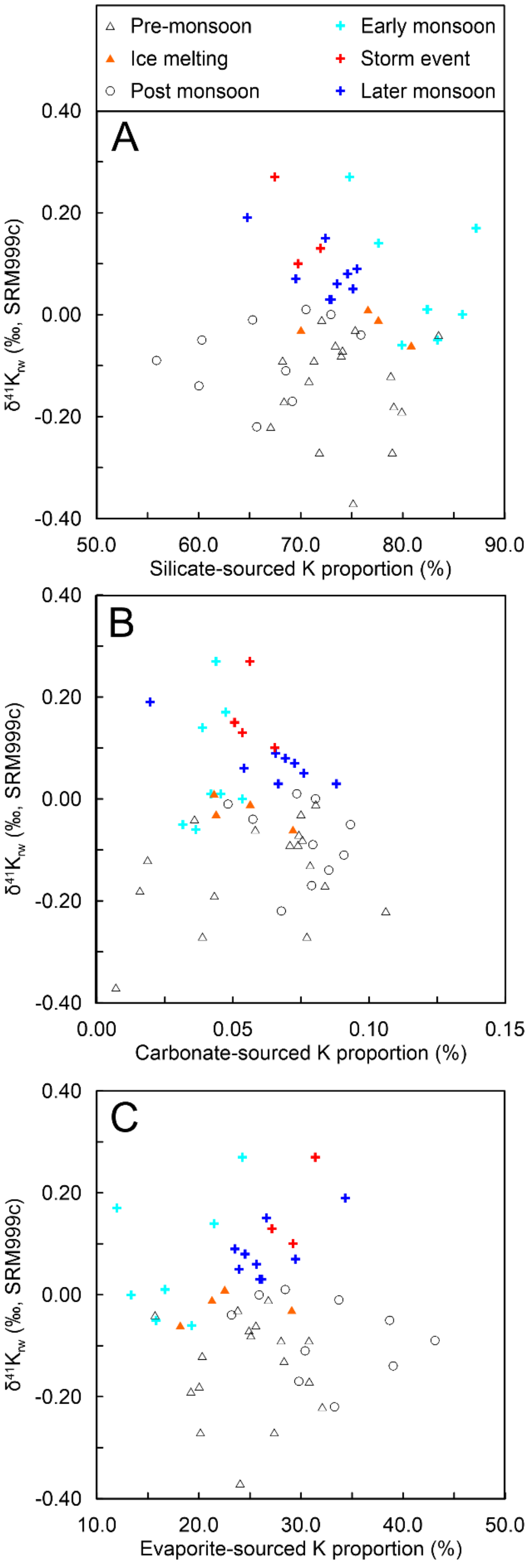

Figure S6 Cross plots between $\delta^{41}K_{rw}$ values and K^+ sourced from (A) silicates, (B) carbonates, and (C) evaporites. The $\delta^{41}K_{rw}$ values appear to be unrelated to any single source contribution.

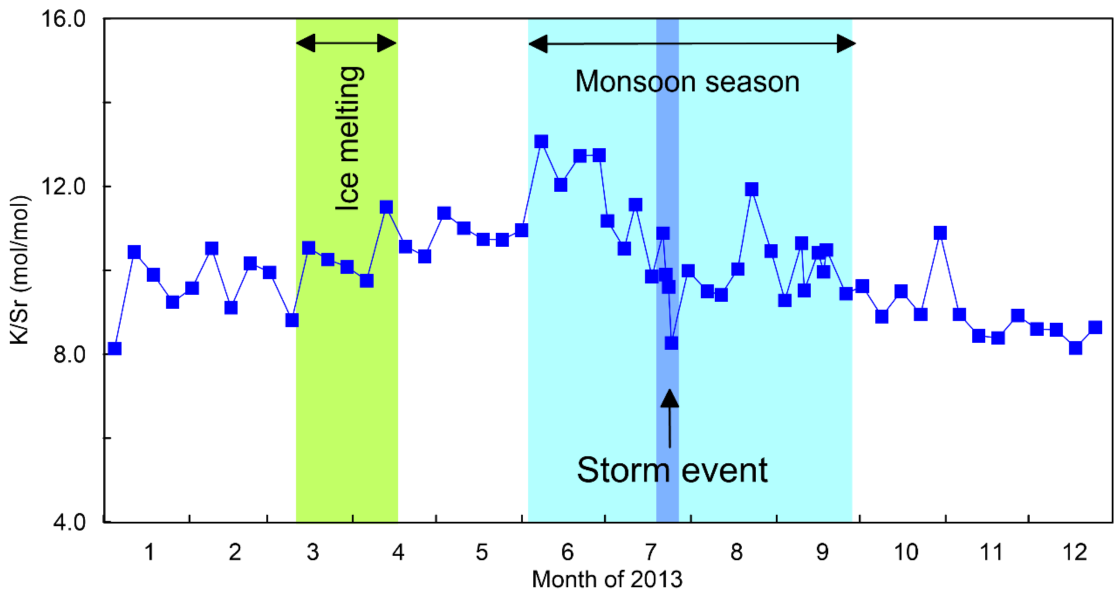

Figure S7 Dissolved K/Sr ratios of the middle Yellow River waters from January to December 2013.

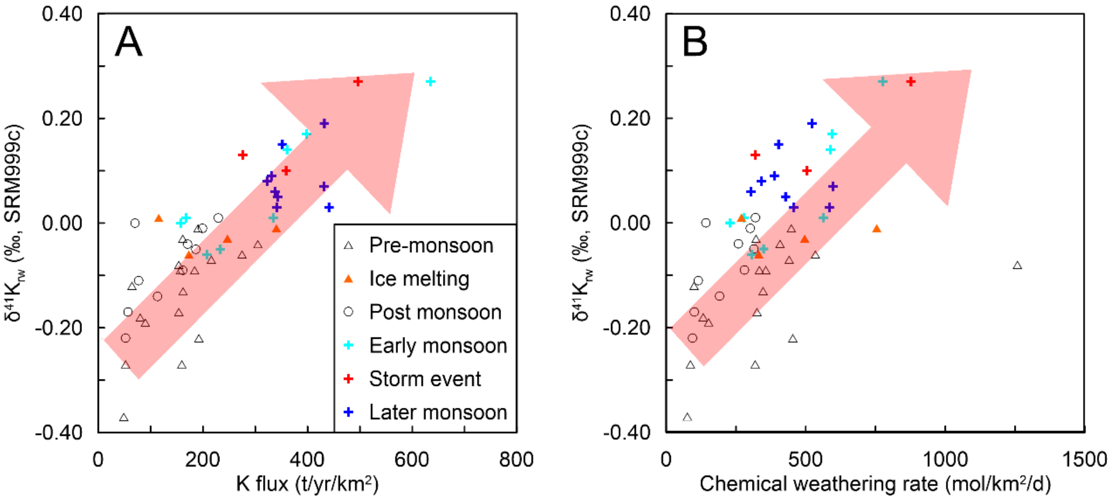

Figure S8 Cross plots of $\delta^{41}K$ values versus (A) dissolved K^+ flux and (B) chemical weathering rate (Zhang et al., 2015)[2], with arrows indicating broad positive correlations.

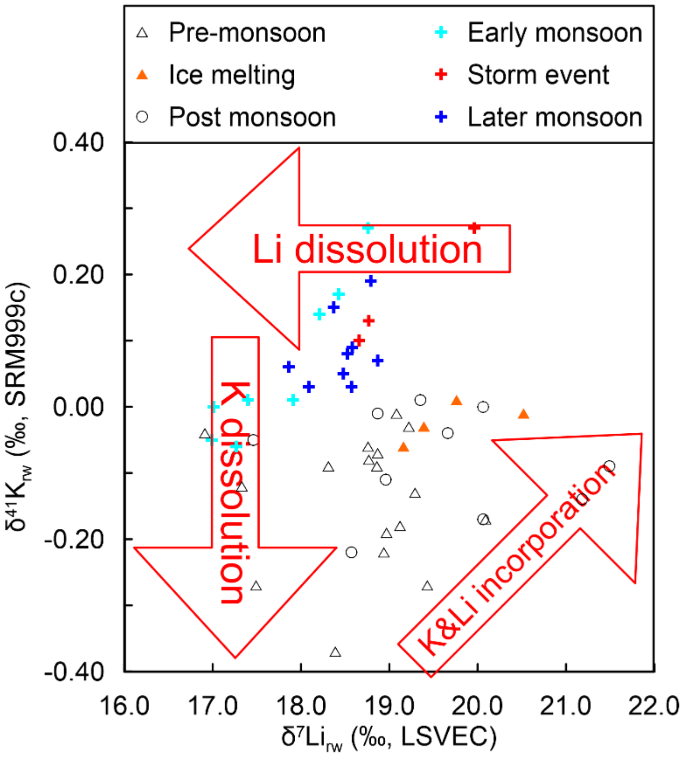

Figure S9 Cross plot between δ^7Li_{rw} and $\delta^{41}K_{rw}$ in the middle Yellow River. The temporal δ^7Li_{rw} variations were shown to result from a temperature dependency[6], which is therefore not driving the $\delta^{41}K_{rw}$ variations. Other processes affecting Li and K isotopes are indicated schematically with arrows.

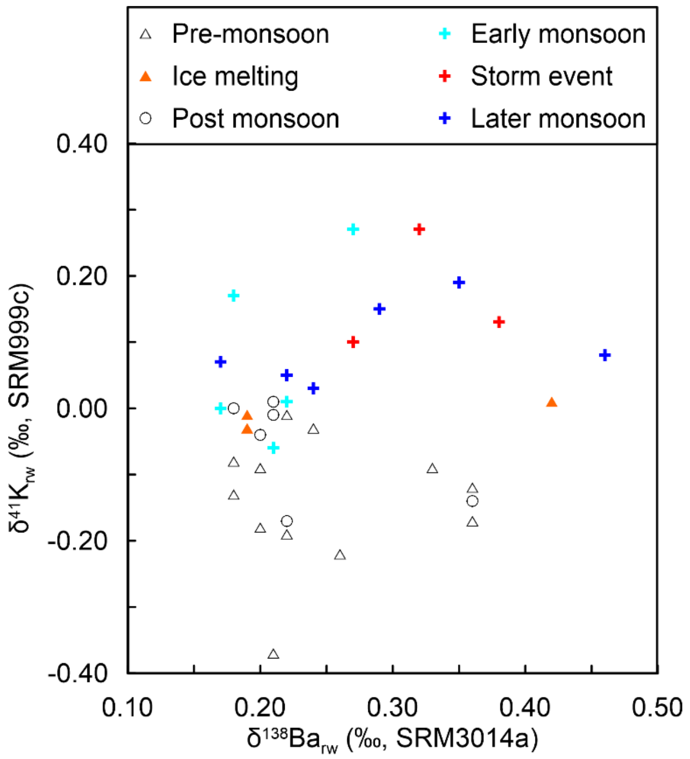

Figure S10 Cross plot between $\delta^{138}Ba_{rw}$ and $\delta^{41}K_{rw}$, with the lack of correlation suggesting that $\delta^{41}K_{rw}$ values are not controlled by adsorption. The $\delta^{138}Ba_{rw}$ data are from Gou et al. (2020) [7].

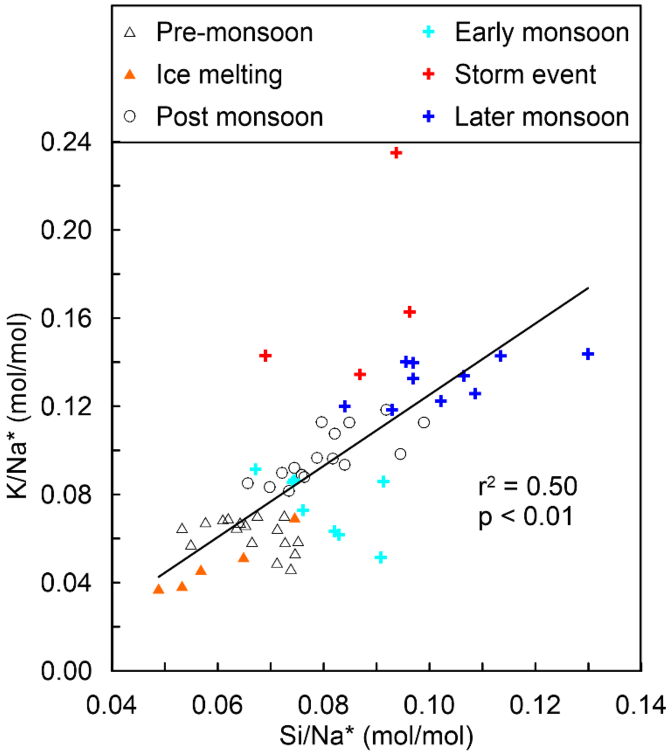

Figure S11 Cross plot between Si and K^+ concentrations, normalized to Na* (Na* = Na^+ - Cl^-). Note the high K/Si during the storm event interval (red crosses), indicating more incongruent silicate weathering, which led to the net release of more K^+ than Si, whereas removal through aluminosilicate neoformation uses at least twice as much Si than K^+ (Table S4).

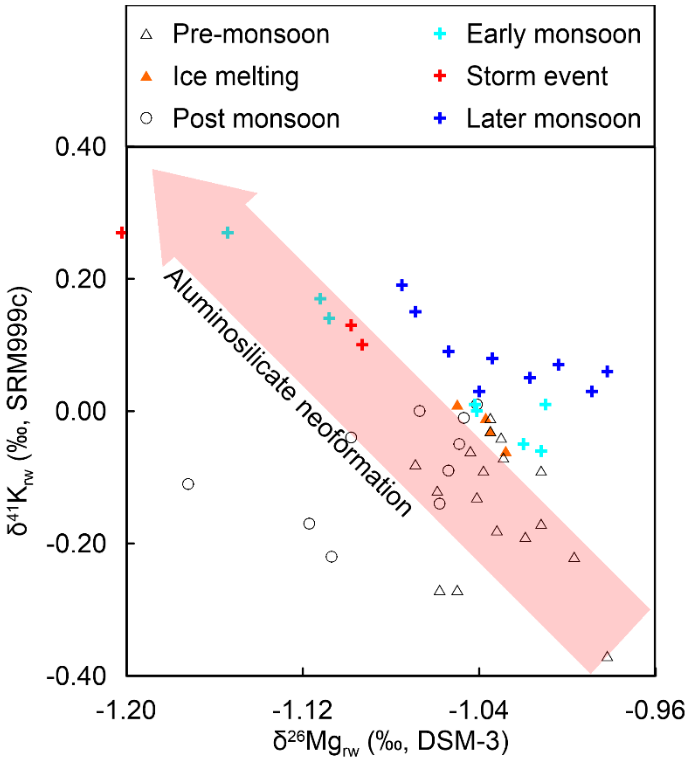

Figure S12 Cross plot between $\delta^{26}Mg_{rw}$ and $\delta^{41}K_{rw}$. The $\delta^{26}Mg_{rw}$ variations are controlled by carbonate dissolution and aluminosilicate neoformation (Gou et al., 2023)[1]. The arrow indicates a weak broad negative co-variation, which may be interpreted as due to aluminosilicate neoformation.

Table S1 K^+ concentrations and K isotope data for the dissolved load and associated parameters in the middle Yellow River.

Sample No.	Date in 2013 (dd-mm)	Discharge [a] (m^3/s)	T [a, b] (°C)	pH [a]	TDS [a, c] (mg/L)	Dissolved load K^+ (umol/L)	$\delta^{41}K_{rw}$ (‰)	2 s.d.[d] (‰)
LM13-1	5-Jan	588.3	0.0	7.64	1063	132	-0.22	0.19
LM13-2	12-Jan	456.4	1.1	7.88	1066	163	-0.09	0.02
LM13-3	19-Jan	468.0	0.1	7.93	905	138	-0.27	0.02
LM13-4	26-Jan	474.8	1.4	7.89	913	131	-0.17	0.13
LM13-5	2-Feb	527.7	1.1	8.06	831	124	-0.13	0.11
LM13-6	9-Feb	518.8	0.6	8.13	766	126	-0.03	0.11
LM13-7	16-Feb	609.1	2.0	8.05	735	104	-0.09	0.13
LM13-8	23-Feb	560.4	1.9	8.02	702	111	-0.08	0.10
LM13-9	2-Mar	832.0	5.0	7.87	671	105	-0.07	0.03
LM13-10	10-Mar	854.5	5.3	7.73	654	90	-0.01	0.12
LM13-11	16-Mar	1110.1	7.1	7.89	629	100	-0.06	0.13
LM13-12	23-Mar	1513.6	7.9	7.93	600	91	-0.01	0.12
LM13-13	30-Mar	402.9	12.5	7.86	768	116	0.01	0.14
LM13-14	6-Apr	845.9	10.7	7.86	806	118	-0.03	0.03
LM13-15	13-Apr	647.8	15.1	8.01	656	119	n.a.	
LM13-16	20-Apr	680.1	11.4	8.01	607	103	-0.06	0.16
LM13-17	27-Apr	338.3	14.5	8.07	627	106	n.a.	
LM13-18	4-May	284.1	15.6	7.94	612	114	-0.18	0.08
LM13-19	11-May	318.7	16.3	8.13	627	114	-0.19	0.05
LM13-20	18-May	167.3	15.0	7.83	635	117	-0.37	0.00
LM13-21	25-May	179.2	16.4	7.78	633	118	-0.27	0.10
LM13-22	1-Jun	225.0	18.6	7.82	614	116	-0.12	0.00
LM13-23	8-Jun	971.8	23.8	7.8	590	127	-0.04	0.03
LM13-24	15-Jun	687.1	23.7	7.86	620	122	-0.06	0.08
LM13-25	22-Jun	773.6	22.7	7.83	576	122	-0.05	0.10
LM13-26	29-Jun	493.2	20.1	7.55	573	129	0.00	0.03
LM13-27	2-Jul	1276.3	27.7	7.99	553	106	0.01	0.01
LM13-28	8-Jul	589.6	27.7	7.83	587	115	0.01	0.01
LM13-29	12-Jul	1353.8	24.2	7.73	617	119	0.17	0.06
LM13-30	18-Jul	1350.9	25.0	7.83	599	108	0.14	0.14
LM13-31	22-Jul	2400.5	23.3	7.66	542	107	0.27	0.09
LM13-32	23-Jul	2207.6	22.3	8.14	527	92	n.a.	
LM13-33	24-Jul	1452.5	26.1	7.05	598	100	0.10	0.15
LM13-34	25-Jul	2255.6	25.4	7.22	603	89	0.27	0.08
LM13-35	31-Jul	990.3	25.6	7.71	671	113	0.13	0.13
LM13-36	7-Aug	1370.0	24.5	8.02	623	104	0.15	0.02
LM13-37	12-Aug	1634.2	26.2	7.92	671	107	0.19	0.11
LM13-38	18-Aug	1106.8	28.8	7.89	658	118	0.08	0.16
LM13-39	23-Aug	1093.3	25.6	7.85	665	125	0.06	0.07

LM13-40	30-Aug	1205.7	23.6	8.30	621	111	0.09	0.04
LM13-41	4-Sep	1392.3	23.2	8.30	622	99	0.03	0.02
LM13-42	10-Sep	1238.6	21.7	8.31	616	112	0.05	0.01
LM13-43	11-Sep	1745.0	21.1	8.30	626	100	0.07	0.09
LM13-44	16-Sep	1715.6	22.0	8.30	583	104	0.03	0.06
LM13-45	18-Sep	1213.0	21.4	8.23	558	98	n.a.	
LM13-46	19-Sep	1723.3	21.1	7.87	548	94	n.a.	
LM13-47	26-Sep	1211.0	17.6	8.71	585	94	n.a.	
LM13-48	2-Oct	901.3	19.3	8.48	652	103	0.01	0.03
LM13-49	9-Oct	797.3	20.2	8.28	693	101	n.a.	
LM13-50	16-Oct	789.6	15.3	8.40	649	102	-0.01	0.04
LM13-51	23-Oct	310.9	11.7	8.32	577	91	0.00	0.07
LM13-52	30-Oct	604.7	11.6	7.72	611	114	-0.04	0.00
LM13-53	6-Nov	312.2	12.3	8.08	647	100	-0.11	0.12
LM13-54	13-Nov	219.8	8.6	8.23	709	104	-0.17	0.06
LM13-55	20-Nov	205.4	5.6	7.32	701	102	-0.22	0.19
LM13-56	27-Nov	573.4	4.3	7.51	741	102	n.a.	
LM13-57	4-Dec	1113.9	3.0	7.70	697	93	n.a.	
LM13-58	11-Dec	712.1	1.7	7.92	807	106	-0.05	0.00
LM13-59	18-Dec	626.7	0.8	7.89	847	104	-0.09	0.10
LM13-60	25-Dec	398.7	0.3	8.30	873	115	-0.14	0.04

[a] Data from Zhang et al. (2015) [2]; see this study for the complete cation data.

[b] T = water temperature.

[c] TDS = total dissolved solids.

[d] “2 s.d.” refers to the two standard deviation from at least triplicate analyses of the same solution.

n.a. = not available, due to samples being exhausted in previous measurements.

Table S2 Major ions and K isotopes of samples of rainwater, agricultural sewage, and groundwater collected from the Longmen hydrological station.

Sample	Type	Date	Na^+	Ca^{2+}	Mg^{2+}	K^+	Cl^-	SO_4^{2-}	NO_3^-	F^-	$\delta^{41}K$	2 s.d. [b]
						μmol/L [a]					‰	‰
LM-r1	rain	8-Jul-2013	244	136	60	27	180	202	323	0.376	-0.68	0.13
LM-r2	rain	14-Jul-2013	134	182	55	45	93	230	225	0.599	n.a.	
LM-r3	rain	17-Jul-2013	78	171	20	17	49	201	78	0.510	n.a.	
TKT1	sewage	12-Jan-2017	902000		12300	827					-0.50	0..03
T10GW	groundwater	26-Jul-2017	15438	1287	3309	62	3570	4528	435	72	-0.05	0.00

[a] Cation and anion data are from Zhang et al. (2015)[2].

[b] "2 s.d." refers to the two-standard deviation from at least triplicate analyses of the same solution.

Table S3 K and Mg concentrations and K isotopic compositions of the sequential extracted loess fractions from Lingtai.

Loess Strata	Extracted Mg [a] (mg/g)	Extracted K (mg/g)	$\delta^{41}K$ (‰)	2 s.d. [b] (‰)	Mean ± 1 s.d. K (mg/g)	Mean ± 1 s.d. $\delta^{41}K$ (‰)
$L1_{evap}$	0.35	0.08	0.18	0.07	**0.14 ± 0.12**	**0.03 ± 0.30**
$S1_{evap}$	0.13	0.07	0.23	0.15		
$S5_{evap}$	0.17	0.07	/	/		
$L9_{evap}$	0.27	0.12	-0.32	0.13		
Rc_{evap}	0.45	0.35	/	/		
$L1_{carb}$	1.51	0.5	-0.30	0.09	**0.62 ± 0.24**	**-0.17 ± 0.08**
$S1_{carb}$	0.63	0.5	-0.17	0.03		
$S5_{carb}$	1.16	0.5	-0.10	0.10		
$L9_{carb}$	0.47	0.6	-0.14	0.07		
Rc_{carb}	1.24	1.0	-0.12	0.02		
$L1_{sil}$	18.80	12.1	-0.28	0.06	**18.29 ± 4.29**	**-0.36 ± 0.12**
$S1_{sil}$	15.76	20.0	-0.25	0.02		
$S5_{sil}$	27.23	21.4	-0.41	0.06		
$L9_{sil}$	22.25	25.9	-0.51	0.05		
Rc_{sil}	20.44	12.0	/	/		

[a] Mg concentration data are from Gou et al. (2023)[1].

[b] "2 s.d." refers to the two-standard deviation from triplicate analyses of the same solution.

Table S4 Saturation indices (SI) of selected aluminosilicates calculated using PHREEQC (version 3, USGS; Parkhurst and Appelo, 1999) [23].

Sample name	Alunite $KAl_3(SO_4)_2(OH)_6$	Illite $K_{0.6}Mg_{0.25}Al_{2.3}Si_{3.5}O_{10}(OH)_2$	Jarosite-K $KFe_3(SO_4)_2(OH)_6$	K-feldspar $KAlSi_3O_8$	K-mica $KAl_3Si_3O_{10}(OH)_2$
LM-1	0.45	-1.14	-6.53	-3.46	6.26
LM-2					
LM-3	-2.71	-2.73	-7.41	-4.07	4.17
LM-4	-2.66	-2.75	-6.50	-4.11	4.14
LM-5	-3.85	-3.13	-6.34	-4.24	3.61
LM-6	-3.44	-2.62	-5.82	-3.99	4.29
LM-7	-4.42	-3.74	-7.45	-4.66	2.91
LM-8	-2.69	-2.55	-5.24	-4.16	4.52
LM-9	-3.70	-3.90	-7.41	-4.96	2.90
LM-10	-2.92	-3.92	-8.04	-5.18	3.00
LM-11	-3.04	-3.74	-5.48	-5.12	3.32
LM-12	-4.58	-4.97	-4.23	-5.80	1.83
LM-13	-5.38	-5.40	-7.23	-5.85	1.12
LM-14	-4.01	-4.37	-7.74	-5.32	2.39
LM-15					
LM-16					
LM-17	-5.89	-4.82	-6.93	-5.50	1.76
LM-18	-7.01	-6.49	-6.99	-6.42	-0.22
LM-19					
LM-20	-5.79	-5.91	-6.52	-6.17	0.53
LM-21					
LM-22	-7.30	-7.22	-8.43	-6.90	-1.03
LM-23	-6.36	-6.48	-8.57	-6.66	0.01
LM-24					
LM-25	-5.86	-5.61	-7.73	-6.11	0.95
LM-26	-3.94	-4.64	-6.20	-5.58	2.13
LM-27	-8.10	-6.53	-9.93	-6.49	-0.35
LM-28	-7.49	-6.21	-8.08	-6.23	-0.05
LM-29	-6.25	-5.60	-7.57	-5.92	0.74
LM-30	-6.93	-5.78	-8.37	-5.95	0.42
LM-31	-1.56	-1.97	-2.43	-4.21	5.33
LM-32	-2.96	-1.07	-2.34	-3.45	6.12
LM-33	-2.06	-4.65	-6.52	-5.87	2.21
LM-34	-3.24	-4.68	-6.76	-5.71	1.95
LM-35	-6.48	-5.77	-6.93	-5.93	0.41
LM-36	-7.20	-5.41	-6.94	-5.69	0.81
LM-37	-6.68	-5.37	-6.58	-5.73	0.89
LM-38	-9.13	-7.31	-8.46	-6.62	-1.56
LM-39	-6.10	-5.29	-6.53	-5.74	1.10

LM-40	-7.52	-4.81	-5.64	-5.30	1.52
LM-41	-6.78	-4.27	-5.55	-5.11	2.23
LM-42	-8.39	-5.56	-7.91	-5.63	0.56
LM-43	-10.58	-7.33	-10.71	-6.44	-1.75
LM-44	-9.00	-6.11	-8.82	-5.93	-0.12
LM-45	-7.37	-5.01	-6.66	-5.47	1.31
LM-46	-6.35	-5.50	-7.60	-5.88	0.84
LM-47	-10.84	-6.12	-9.50	-5.64	-0.37
LM-48	-9.15	-5.61	-8.51	-5.54	0.38
LM-49	-7.84	-5.40	-7.27	-5.61	0.79
LM-50					
LM-51	-7.32	-4.88	-8.61	-5.26	1.41
LM-52	-3.47	-4.01	-6.33	-5.11	2.81
LM-53	-5.96	-4.81	-6.71	-5.36	1.63
LM-54	-5.95	-4.17	-6.77	-4.85	2.28
LM-55					
LM-56	-1.29	-3.29	-8.48	-4.84	3.79
LM-57	-1.87	-3.09	-7.35	-4.64	3.95
LM-58					
LM-59	-1.15	-1.95	-5.23	-3.94	5.27
LM-60	-4.53	-3.05	-6.52	-4.11	3.62

Values > 0 imply supersaturation.

Note that samples LM 31-34 correspond to the storm event.

Supplementary references

1. Gou L-F, Jin Z, Galy A, Xu Y, Xiao J, Yang Y, Bouchez J, Pogge von Strandmann PAE, Jin C, Yang S, Zhao Z-Q. Seasonal Mg isotopic variation in the middle Yellow River: Sources and fractionation. *Chemical Geology* 619, 121314 (2023).

2. Zhang Q, Jin Z, Zhang F, Xiao J. Seasonal variation in river water chemistry of the middle reaches of the Yellow River and its controlling factors. *Journal of Geochemical Exploration* **156**, 101-113 (2015).

3. Li G, West AJ. Evolution of Cenozoic seawater lithium isotopes: Coupling of global denudation regime and shifting seawater sinks. *Earth and Planetary Science Letters* **401**, 284-293 (2014).

4. Gaillardet J, Dupre B, Louvat P, Allegre CJ. Global silicate weathering and CO_2 consumption rates deduced from the chemistry of large rivers. *Chemical Geology* **159**, 3-30 (1999).

5. Gaillardet J, Viers J, Dupré B. 7.7 - Trace Elements in River Waters A2 - Holland, Heinrich D. In: *Treatise on Geochemistry (Second Edition)* (ed Turekian KK). Elsevier (2014).

6. Gou L-F, Jin Z, Pogge von Strandmann PAE, Li G, Qu Y-X, Xiao J, Deng L, Galy A. Lithium isotopes in the middle Yellow River: Seasonal variability, sources and fractionation. *Geochimica et Cosmochimica Acta* **248**, 88-108 (2019).

7. Gou L-F, Jin Z, Galy A, Gong Y-Z, Nan X-Y, Jin C, Wang X-D, Bouchez J, Cai H-M, Chen J-B, Yu H-M, Huang F. Seasonal riverine barium isotopic variation in the middle Yellow River: Sources and fractionation. *Earth and Planetary Science Letters* **531**, 115990 (2020).

8. Wang K, Close HG, Tuller-Ross B, Chen H. Global average potassium isotope composition of modern seawater. *ACS Earth and Space Chemistry* **4**, 1010-1017 (2020).

9. Huang T-Y, Teng F-Z, Rudnick LR, Chen X-Y, Hu Y, Liu YS, Wu F-Y. Heterogeneous potassium isotopic composition of the upper continental crust. *Geochimica et Cosmochimica Acta* **278**, 122-136 (2020).

10. Jiang Y, Koefoed P, Pravdivtseva O, Chen H, Li C-H, Huang F, Qin L-P, Liu J, Wang K, Early solar system aqueous activity: K isotope evidence from Allende. *Meteoritics & Planetary Science* **56**, 61-76 (2020).

11. Hu Y, Teng F-Z, Plank T, Chauvel C. Potassium isotopic heterogeneity in subducting

oceanic plates. *Science Advances* **6**, eabb2472 (2020).

12. Santiago Ramos DP, Coogan LA, Murphy JG, Higgins JA. Low-temperature oceanic crust alteration and the isotopic budgets of potassium and magnesium in seawater. *Earth and Planetary Science Letters* **541**, 116290 (2020).

13. Xu Y-K, Hu Y, Chen X-Y, Huang T-Y. Sletten RS, Zhu D, Teng F-Z. Potassium isotopic compositions of international geological reference materials. *Chemical Geology* **513**, 101-107 (2019).

14. Hu Y, Chen XY, Xu YK, Teng FZ. High-precision analysis of potassium isotopes by HR-MC-ICPMS. *Chemical Geology* **493**, 100-108 (2018).

15. Li X, Han G, Zhang Q, Miao Z. An optimal separation method for high-precision K isotope analysis by using MC-ICP-MS with a dummy bucket. *Journal of Analytical Atomtic Spectrometry* **35**, 1330-1339 (2020).

16. Li S, Li W, Raymo ME, Wang X, Chen Y, Chen J. K isotopes as a tracer for continental weathering and geological K cycling. *Proceedings of the National Academy of Sciences of the United States of America* **116**, 8740-8745 (2019).

17. Ji T-T, Jiang X-W, Han G, Li X, Wan L, Wang Z-Z, Guo H, Jin Z. Contrasting behavior of K isotopes in modern and fossil groundwater: Implications for K cycle and subsurface weathering. *Earth and Planetary Science Letters* **626**, 118526 (2024).

18. Li X, Han G, Zhang Q, Liu J, Qu R. Contrasting riverine K and Li isotope signatures during silicate weathering in the southeastern Tibetan Plateau. *Earth and Planetary Science Letters* **622**, 118402 (2023).

19. Wang K, Peucker-Ehrenbrink B, Chen H, Lee H, Hasenmueller EA. Dissolved potassium isotopic composition of major world rivers. *Geochimica et Cosmochimica Acta* **294**, 145-159 (2021).

20. Li X, Han G, Liu M, Liu J, Zhang Q, Qu R. Potassium and its isotope behaviour during chemical weathering in a tropical catchment affected by evaporite dissolution. *Geochimica et Cosmochimica Acta* **316**, 105-121 (2022).

21. Morgan LE, Santiago Ramos DP, Davidheiser-Kroll B, Faithfull J, Lloyd NS, Ellamf RM, Higgins JA. High-precision $^{41}K/^{39}K$ measurements by MC-ICP-MS indicate terrestrial variability of $\delta^{41}K$. *Journal of Analytical Atomtic Spectrometry* **33**, 175-186 (2018).

22. Zhao C, Lodders K, Bloom H, Chen H, Tian Z, Koefoed P, Pető MK, Wang K. Potassium isotopic compositions of enstatite meteorites. *Meteoritics & Planetary Science* **55**, 1404-1417 (2020).

23. Parkhurst DL, Appelo CAJ. User's Guide to PHREEQC (Version 2) — A computer program for speciation, batch-reaction, one-dimensional transport, and inverse geochemical calculations. (1999).
